# Hypocalcemia in combination with hyperphosphatemia impairs muscle cell differentiation in vitro

**DOI:** 10.1007/s40618-023-02212-2

**Published:** 2023-10-11

**Authors:** V. M. Bimonte, G. Catanzaro, Z. Spinello, M. C. Massari, M. Curreli, G. Terrana, G. Defeudis, J. Halupczok-Żyła, G. Mantovani, E. Ferretti, S. Migliaccio

**Affiliations:** 1 Department of Movement, Human and Health Sciences, University of Foro Italico, Largo Lauro De Bosis 6, 00195 Rome, Italy; 2 Department of Experimental Medicine, University “Sapienza” of Rome, 00161 Rome, Italy; 3 Department of Endocrinology, Diabetes and Isotope Therapy, Wroclaw Medical University, 50004 Wrocław, Poland; 4 Department of Clinical Sciences and Community Health, University of Milan, 20122 Milan, Italy; 5 Endocrinology Unit, Fondazione IRCCS Ca’ Granda Ospedale Maggiore Policlinico, 20122 Milan, Italy

**Keywords:** Hypoparathyroidism, Hyperphosphatemia, Hypocalcemia, Skeletal muscle cells, Myotube, Insulin-like growth factor-1

## Abstract

**Purpose:**

Hypoparathyroidism is a rare endocrine disorder characterized by low or absent secretion of parathyroid hormone (PTH), which leads to decreased calcium and increased phosphorus levels in the serum. The diagnosis of hypoparathyroidism is based on the identification of the aforementioned biochemical abnormalities, which may be accompanied by clinical manifestations. Symptoms of hypoparathyroidism, primarily attributed to hypocalcemia, include muscle cramps or spasms, facial, leg, and foot pain, seizures, and tingling in the lips or fingers. The treatment of hypoparathyroidism depends on the severity of symptoms and the underlying pathology. Over the long term, calcium supplements, active vitamin D analogs, and thiazide diuretics may be needed. In fact, in patient cohorts in which optimal disease control still remains elusive, replacement therapy with recombinant parathyroid hormone analogs may be contemplated. Despite the predominantly neuromuscular symptoms of hypoparathyroidism, further effects of parathyroid hormone deficiency at the muscle cell level remain poorly understood. Thus, the aim of our study was to evaluate the effects of hypocalcemia in combination with hyperphosphatemia on muscle cells differentiation in vitro.

**Methods:**

C2C12 cells, an in vitro model of muscle cells, were differentiated for 2 or 6 days in the presence of hypocalcemia (CaCl_2_ 0.9 mmol/l) and moderate (PO4 1.4 mmol/l) or severe (PO4 2.9 mmol/l) hyperphosphatemia, or combinations of both conditions. Cell differentiation and expression of genes linked to muscle differentiation were evaluated.

**Results:**

The combination of hypocalcemia with hyperphosphatemia induced a significant reduction (50%) in differentiation marker levels, such as MyoD (protein 1 for myoblast determination) and myogenin on the 1st day of differentiation, and MHC (myosin heavy chains) after 6 days of differentiation compared to control. Furthermore, this condition induced a statistically significant reduction of insulin-like growth factor-1 (IGF-1) mRNA expression and inhibition of IGF signaling and decrease in ERK phosphorylation compared to control cells.

**Conclusions:**

Our results showed that a condition of hypocalcemia with hyperphosphatemia induced an alteration of muscle cell differentiation in vitro. In particular, we observed the reduction of myogenic differentiation markers, IGF-1 signaling pathway, and ERK phosphorylation in differentiated skeletal myoblasts. These data suggest that this altered extracellular condition might contribute to the mechanisms causing persistence of symptoms in patients affected by hypoparathyroidism.

## Introduction

Hypoparathyroidism is a rare endocrine disorder caused by the reduced or absent production of parathyroid hormone (PTH) by the parathyroid glands, which leads to low calcium and high phosphate serum concentrations [1, 2]. Several different factors can contribute to hypoparathyroidism, such as surgical removal of the parathyroid glands, autoimmune dysfunction, and genetic and infiltrative diseases [1, 2]. The main cause of hypoparathyroidism is damage of the parathyroid glands during surgery with an incidence rate ranging from 1 to 7% in patients undergoing thyroidectomy [3]. Clinical symptoms of acute hypoparathyroidism are consequence of hypocalcemia and they are mainly neuromuscular: cramps, paresthesia, muscle contractions, and in severe cases medical emergencies, such as tetany and bronchospasm [4, 5]. Although the therapy of acute hypocalcaemia is usually readily accomplished, chronic hypocalcemia still remains difficult to treat, with any definitive treatment. Anyway, replacement therapy with recombinant PTH analogs could be a therapeutic option for refractory HPT [6].

The chronic effects of hypoparathyroidism have been studied mainly in bone tissue, and they are characterized by alteration of bone remodeling, and reduced bone mass, causing osteoporosis with an increased risk of fractures [7, 8]. On the other hand, although the muscle is particularly affected by hypocalcemia due to the lack of PTH, the effects of hypoparathyroidism at the muscle level are less known and characterized.

The literature presents various indirect associations between parathyroid, bone, and muscle. First, it has been observed in clonal cell lines of mouse myoblasts that the active form of vitamin D, or 1,25-dihydroxyvitamin D, which is largely produced due to the action of PTH on 1-alpha hydroxylase, reduces the expression of myostatin mRNA, a muscle cytokine involved in the inhibition of muscle growth and age-related sarcopenia. This suggests possible effects of calcitriol on muscle trophism [5]. Furthermore, a randomized controlled trial in patients with hypoparathyroidism treated with recombinant PTH versus placebo showed that the PTH-treated group had a significant increase in (decarboxylated) osteocalcin. PTH promotes the synthesis of this molecule in osteoblasts and recent data indicate that osteocalcin plays an important role in muscle metabolism, likely regulating muscle mass and glucose uptake [9]. However, human studies on (decarboxylated) osteocalcin are inconclusive and currently at discordant with animal model studies.

Further, osteocalcin is also involved in the increase of circulating levels of interleukin 6 that represent of the main ones proinflammatory cytokine. This cytokine is produced and released by muscle in response to physical exercise that it also has among its targets skeletal muscle itself [10]. At this level, it stimulates myogenesis and muscle trophism and partly regulates energy metabolism [10]. The hypothesis that muscle may be a direct target of PTH is supported by the fact that both of its receptors (PTHR1 and PTHR2) are expressed in skeletal muscle on muscle fibers and mature myotubes [11].

Direct effects have also been hypothesized in an in vivo study on murine models with muscle atrophy and osteoporosis, where treatment with recombinant PTH 1–34 seems to increase femoral bone mineral density (BMD) and the percentage of muscle mass [12]. Another in vivo study showed the action of PTH on myogenesis, acting on the differentiation of myocytes and accelerating the formation of myotubes [13].

Another hormone with anabolic actions on various tissues is the insulin-like growth factor-1 (IGF-1), which is produced mainly in the liver in response to growth hormone (GH) stimulation. IGF-1 acts by binding to its receptor (IGF1R) on the surface of various tissues activating numerous cellular processes that promote cell growth, proliferation, and differentiation leading to protein synthesis and contributing to increased muscle mass and strength.

Few data suggest that PTH and IGF-1 may have a synergistic action on bone through the activation of the same receptor, PTH1R, which can be phosphorylated by IGF1R, leading to the differentiation of osteoblasts into osteocytes [14]. Further evidence of a correlation between the two hormones has been found in studies in animal models. Intermittent administration of PTH in mice KO for IGF-1 and IGF1R showed that the anabolic effect of PTH on bone was reduced, suggesting that PTH action is, at least in part, mediated by the IGF1–IGF1R axis [15]. In contrast, no studies have evaluated the potential interaction between PTH and IGF-1 on muscle, even though IGF-1 deficiency leads to hypotrophy, reduced muscle strength, and impaired anaerobic exercise capacity.

Despite all these data, the effects and consequences of hypoparathyroidism in human skeletal muscle remain largely unexplored, both in in vitro and clinical studies. Nevertheless, few studies have evaluated whether the alteration of serum ions, calcium and phosphate modulated by PTH, might also acts directly on muscle cells affecting differentiation process and homeostasis. To address this issue, our study aimed to create an in vitro model resembling hypoparathyroidism ionic condition to evaluate the effects of low calcium in combination with high inorganic phosphate on the differentiation and biology of myoblast cells. Furthermore, we investigated whether such altered ions condition that mimic hypoparathyroidism could also lead to changes in IGF-1 expression and signaling in the muscle cells.

## Materials and methods

### Cell culture

Skeletal muscle cells (C2C12) were purchased from LGC Standards (Milan, Italy) and were grown in Dulbecco’s modified Eagle’s medium with high glucose (DMEM) supplemented with 10% fetal bovine serum (FBS), 2 mM glutamine, 100 units/mL penicillin and 0.1 mg/mL streptomycin. Adherent cells were detached by Trypsin–EDTA solution. Cells were maintained at 37 °C in a humidified atmosphere of 5% CO_2_ and 95% air. Differentiation into myotubes was achieved by culturing C2C12 myoblasts in a complete medium and then switching into DMEM medium supplemented with 2% FBS for 1, 3, and 6 days when cells were 90% confluent.

Buffers and reagents for cell culture were purchased from Corning (New York, USA) and medium from PAN-Biotech (Aidenbach, Germany).

### Treatments protocols

In all experiments, cells were seeded at the density of 10^4^ cells/cm^2^ and allowed to grow until 90% confluence in culture media containing: DMEM with 0.5 mM phosphate (moderate hyperphosphatemia- PO4 0.5 mM) or 2 mM phosphate (severe hyperphosphatemia- PO4 2 mM) resulted in effectively 1.42 mM and 2.92 mM phosphate [16], DMEM with low calcium concentration (Hypo, CaCl_2_ 0.9 mmol/L), and the combination of hypocalcemia and hyperphosphatemia. The same conditions were also maintained during the differentiation, where fetal bovine serum content was lowered to 2%, when cells were 90% confluent. Different phosphate conditions were prepared by supplementation of a 67:33 mixture of 1 M Na_2_HPO_4_ and 1 M NaH_2_PO_4_ (Sigma-Aldrich, St. Louis, Missouri, USA).

### Protein extraction and Western immunoblotting

Cells were lysed as previously described [17]. For the immunoblot analysis, 15 µg of proteins were separated on 8–12% SDS–polyacrylamide gels and immunoblotted using standard protocols. Primary antibodies and concentrations were used as follows: myosin heavy chains (MHC), extracellular signal-regulated kinase (ERK), pERK1/2 (Thr 44/42) from Santa Cruz Biotechnology; p70 S6 kinase (p70^S6K)^, pp70^S6K^; glycogen synthase kinase 3β (GSK3β), pGSK3βfrom Cell Signaling Technology. Vinculin (1:1000, Cell Signaling Technology Inc.) was used as a loading control. Secondary antibodies were purchased from Jackson Laboratories (Bar Harbor, ME, USA; dilution 1:10.000). Proteins were revealed by enhanced chemiluminescence (LiteAblot^®^ TURBO; EuroClone). Images were acquired with a ChemiDoc Touch Imaging System (Bio‐Rad). Densitometric analysis of the bands was performed using Image J Software v1.51 (NIH, Bethesda, MD, USA).

### RNA isolation and qRT-PCR

RNA was isolated as previously described [18], following the manufacturer’s instructions. Quantitative real-time PCR was performed in ViiA 7 Real-Time PCR (Applied Biosystem, Waltham, MA, USA), using power SYBR green PCR master mix (Aurogene, Rome, Italy), as indicated by the manufacturers. The sequences of the utilized primers were as follows:

**Table:** 

Gene name	Primers
MyHCI	F: CTCAAGCTGCTCAGCAATCTATTT R: GGAGCGCAAGTTTGTCATAAGT
MyHC IIa	F: AGGCGGCTGAGGAGCACGTA R: GCGGCACAAGCAGCGTTGG
MyHC IIb	F: CAATCAGGAACCTTCGGAACAC R: GTCCTGGCCTCTGAGAGCAT
IGFR1	F: GTGGGGGCTCGTGTTTCTC R: GATCACCGTGCAGTTTTCCA
Myf5	F: TGAGGGAACAGGTGGAGAAC R: AGCTGGACACGGAGCAGCTTTTA
IGF-1	F: CAGCTGTTTCCTGTCTACAGTG R: CCTGCACTTCCTCTACTTGTG
GAPDH	F: AACATCAAATGGGGTGACGCC R:GTTGTCATGGATGACCTTGGC
MyoD	F:GGCCTGCAAGGCGTGCAAGC R:GCGTTGCGCAGGATCTCCAC
Miogenin	F: CGATCTCCGCTACAGAGGC R: GTTGGGACCGAACTCCAGT
FNDC5	F:TTGCCATCTCTCAGCAGAAGA R:GGCCTGCACATGGACGATA

All values were normalized to endogenous controls GAPDH

### Immunofluorescence and myogenic differentiation assays

After 6 days of differentiation, cells were washed twice with cold PBS, fixed with 4% formaldehyde for 10 min, permeabilized with bovine serum albumin (BSA) 1%, 0.2% Triton X-100 in PBS for 15 min, and incubated in blocking solution (6 ng/ml IgG from goat serum in PBS) for 30 min. Cells were incubated with anti-MHC antibody (1:200, Santa Cruz Biotechnology) overnight at 4 °C in BSA 3%. Alexa Fluor 488-conjugated anti-rabbit IgG (H + L) was used as secondary antibody (1:500, Thermo Fisher Scientific) in BSA 3% and incubated for 45 min.

Counterstaining was performed with 4′,6-diamidino-2-phenylindole dihydrochloride (DAPI; 500 ng/ml) for 10 min. After three washes in PBS, samples were mounted with Prolong Gold Antifade Reagent (Invitrogen) and analyzed with confocal microscopy (Olympus). Fusion index and differentiation index were measured manually according to Aversa et al. [19].

### Statistical analysis

Data are presented as the mean of standard error (SEM), determined from three or more experiments per condition. Differences between pairs of groups were analyzed by one-way ANOVA followed by a Dunnett post hoc test (GraphPad Prism, San Diego, CA, USA). The level of significance was set as *p < 0.05, **p < 0.01, and ***p < 0.001.

## Results

### Low calcium and hyperphosphatemia conditions modulate myogenic differentiation in C2C12 skeletal muscle cells in vitro

The calcium concentration in the medium was halved (0.9 mM-HypoC), as compared to the normal calcium concentration of a control cell culture medium (1.8 mM), while the concentrations of inorganic phosphate (PO4) for in vitro experiments, (1.42–2.92 mM PO4) were chosen based on previous published data [16].

To evaluate the potential effect of low calcium and high PO4 concentrations on the muscle cells differentiation program, cells were incubated with low calcium in combination of increasing concentrations of PO4. Key myogenic differentiation markers, such as MyoD, myogenin and MHC were analyzed after 1–3–6 days of incubation by qRT-PCR and western blot analyses.

**Fig. 1:**
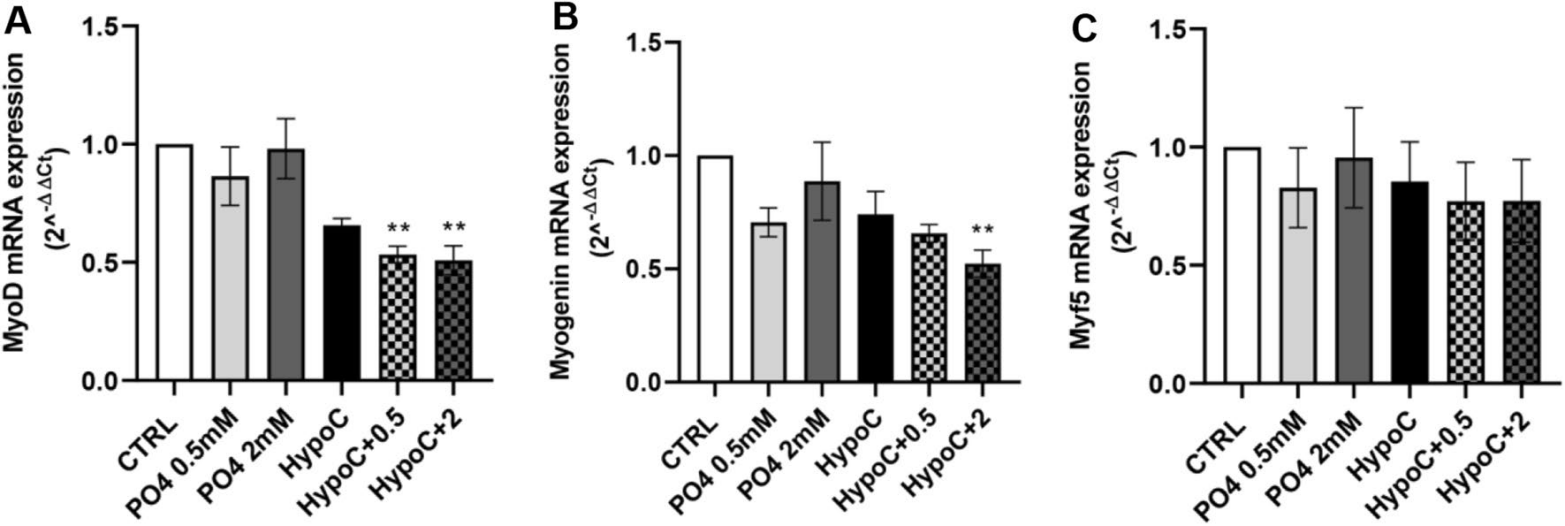
Effect of hypocalcemia and hyperphosphatemia on early muscle differentiation. C2C12 muscle cells were induced to differentiate in the presence or absence of calcium (0.9 mM- 1.8 mM) and different concentrations of P04 (0.5–2 mM). Analysis of gene expression levels by RT-qPCR of the main early markers of muscle differentiation after 1 day of differentiation: **A** MyoD, **B** myogenin and **C** Myf5. GAPDH was used as an internal control (**p < 0.01 vs. CTRL)

As shown in Fig. 1, HypoC + 2 mM PO4 condition induced a statistically significant reduction in the gene expression levels of early differentiation markers, such as Myo- (0.51 ± 0.03-fold decrease; Fig. 1A) and myogenin (0.52 ± 0.06-fold decrease; Fig. 1B) on the first day of differentiation (D1), compared to cells grown under control conditions (CTRL). Moreover, as shown in Fig. 1C, there was no modulation of the myogenic factor 5 (Myf5) mRNA expression, as compared to the control cells.

**Fig. 2:**
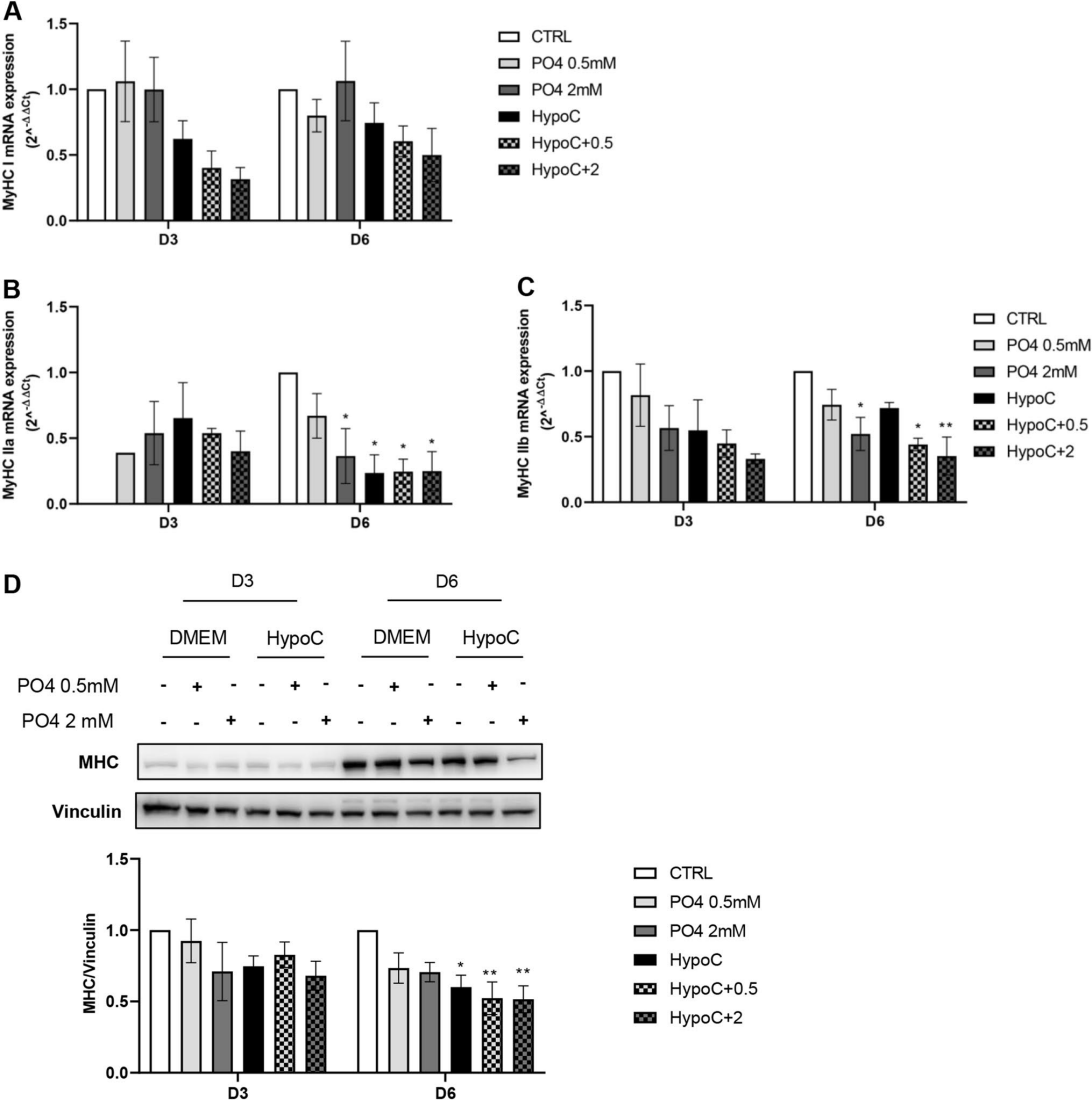
Effect of hypocalcemia and hyperphosphatemia on late muscle differentiation. C2C12 muscle cells were induced to differentiate in the presence or absence of calcium (0.9–1.8 mM) and different concentrations of P04 (0.5–2 mM). Analysis of gene expression levels by RT-qPCR was performed after 3–6 days of differentiation: **A** MyHC I, **B** MyHC IIa, **C** MyHC IIb. Analysis of protein expression of late marker of muscle differentiation by Western blot (D) MHC. GAPDH or vinculin was used as an internal control. (*p < 0.05; **p < 0.01 vs*.* CTRL)

Additionally, as shown in Fig. 2A, there was a trend of decrease of myosin type one (MyHC I), and a statistically significant reduction of myosin type IIa (MyHC IIa) (Fig. 2B) and IIb (MyHC IIb) (Fig. 2C). In particular, the PO4 2 mM condition, the HypoC condition, and the HypoC + PO4 (0.5–2 mM) condition all induced a significant decrease in MyHC IIa mRNA expression compared to CTRL. Furthermore, the PO4 2 mM condition and the HypoC + PO4 (0.5 and 2 mM) condition induced a significant reduction in MyHC IIb mRNA expression compared to CTRL.

Moreover, the HypoC + PO4 (0.5–2 mM) condition induced a statistically significant reduction in protein expression of the late differentiation marker MHC 0.5 ± 0.1-fold at both concentrations, as compared to control cells after 6 days of differentiation (Fig. 2D).

**Fig. 3:**
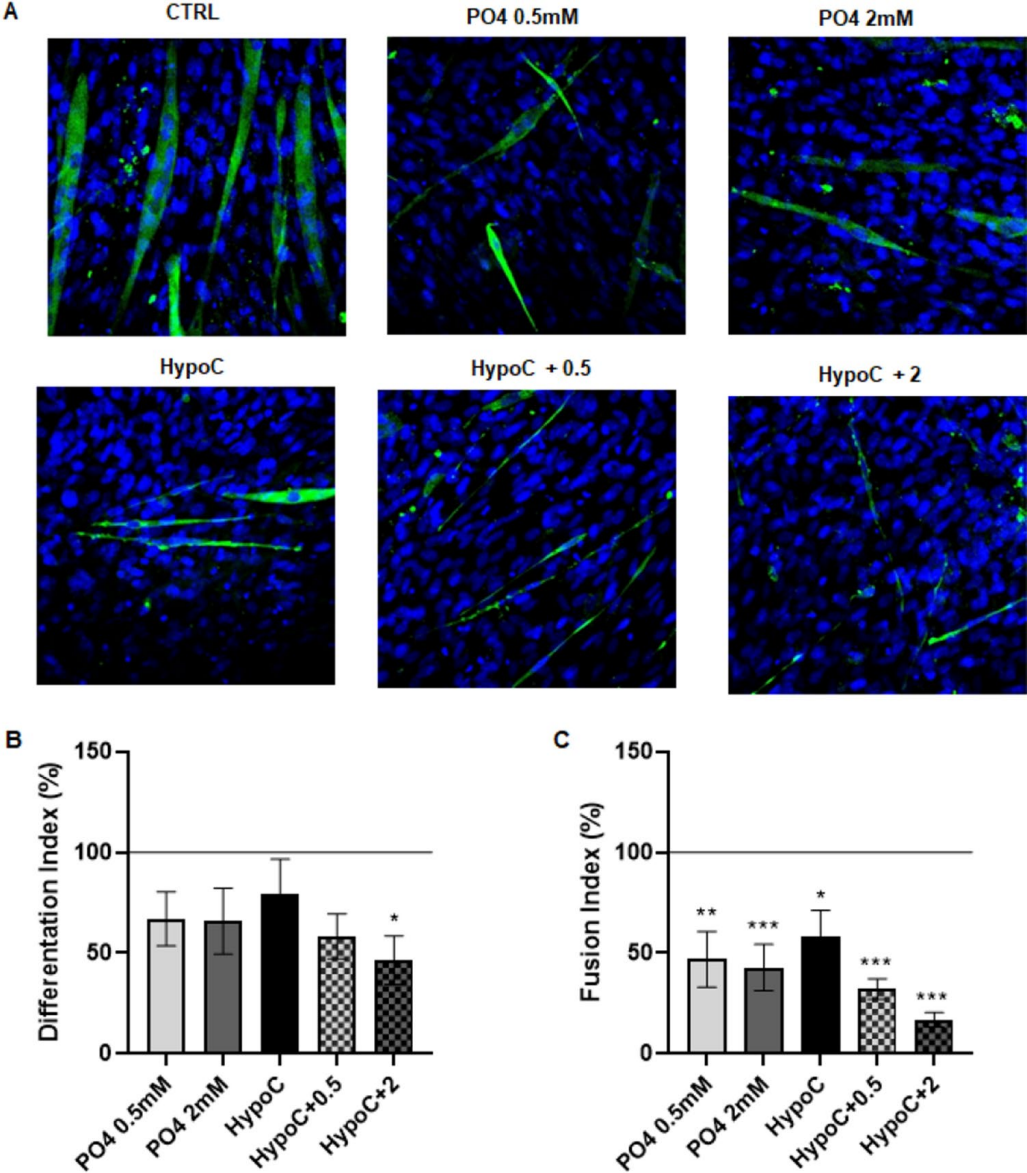
Effect of hypocalcemia and hyperphosphatemia on muscle cell differentiation and myotube formation in vitro. C2C12 cells were differentiated in the presence or absence of calcium (0.9–1.8 mM) and different concentrations of P04 (0.5–2 mM). The figure depicts representative images of immunofluorescence staining for MHC after 6 days of differentiation. Differentiation A) and fusion indexes B) in treated cells were calculated as described in “Materials and methods”. Magnification 20 × . *p < 0.05; **p < 0.01, ***p < 0.001vs*.* CTRL

To confirm that an extracellular condition of hypocalcemia and hyperphosphatemia, as described above) impaired the muscle differentiation in vitro, we performed an immunofluorescence analysis (Fig. 3A). The differentiation index significantly decreased after 6 days in an extracellular hypocalcemia and severe hyperphosphatemia condition (Fig. 3B). Moreover, fusion index for myotubes formation in the differentiated cells decreased after 6 days by ~ 50% and 35% in extracellular hyperphosphatemia and hypocalcemia, respectively. Furthermore, the fusion index decreased by about 70% and 85% in cells cultured with hypocalcemia in combination with moderate and severe hyperphosphatemia conditions, respectively (Fig. 3C).

### Effects of extracellular hypocalcemia and hyperphosphatemia conditions on IGF-1 and irisin expression

**Fig. 4:**
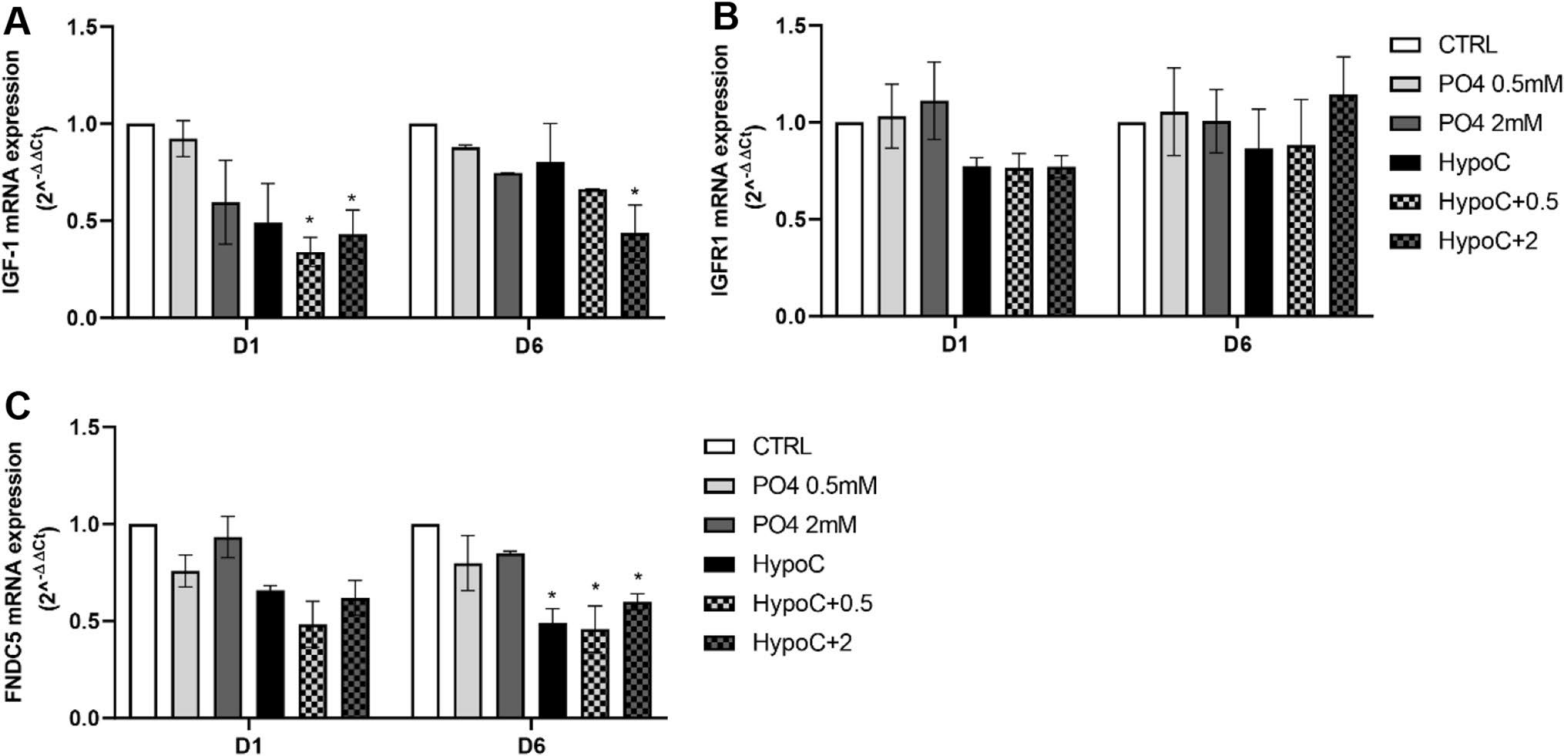
Effect of hypocalcemia and hyperphosphatemia on IGF-1 and FNDC5 in vitro. Analysis of gene expression levels by RT-qPCR of IGF-1 (**A**), IGFR1 (**B**), FNDC5 (**C**) after 1–6 days of differentiation. GAPDH was used as an internal control (*p < 0.05 vs*.* CTRL)

Since several data in literature suggested a role of IGF-1 in muscle cells homeostasis [20–24], we asked whether extracellular hypocalcemia and hyperphosphatemia could alter this growth factor expression level. Interestingly, HypoC in combination with 0.5–2 mM PO4 concentration induced a significant reduction (0.4 ± 0.04 and 0.5 ± 0.12-fold) in IGF-1 mRNA expression after 1 day of differentiation, while 2 mM PO4 induced a significant reduction (0.35 ± 0.14-fold) after 6 days of differentiation (Fig. 3A), without changes in IGFR1 mRNA expression (Fig. 4B), as compared to control cells.

Moreover, we evaluated modification of irisin, a myokine derived from the FNDC5 (fibronectin type III domain-containing protein 5) protein cleavage, with different physiological functions, such as modulation of energy expenditure, glucose/lipid metabolism, and muscle development [25]. Interestingly, HypoC associated with hyperphosphatemia induced a 0.4 ± 0.08-fold decrease in FNDC5 mRNA expression (Fig. 4C), and HypoC in combination with 0.5 or 2 mM PO4 concentration induced a 0.5 ± 0.1 and 0.6 ± 0.05-fold decrease in FNDC5 mRNA expression compared to control, suggesting a detrimental effect on muscle cells.

### Effects of extracellular low calcium and hyperphosphatemia concentration on IGF-1 signaling

**Fig. 5:**
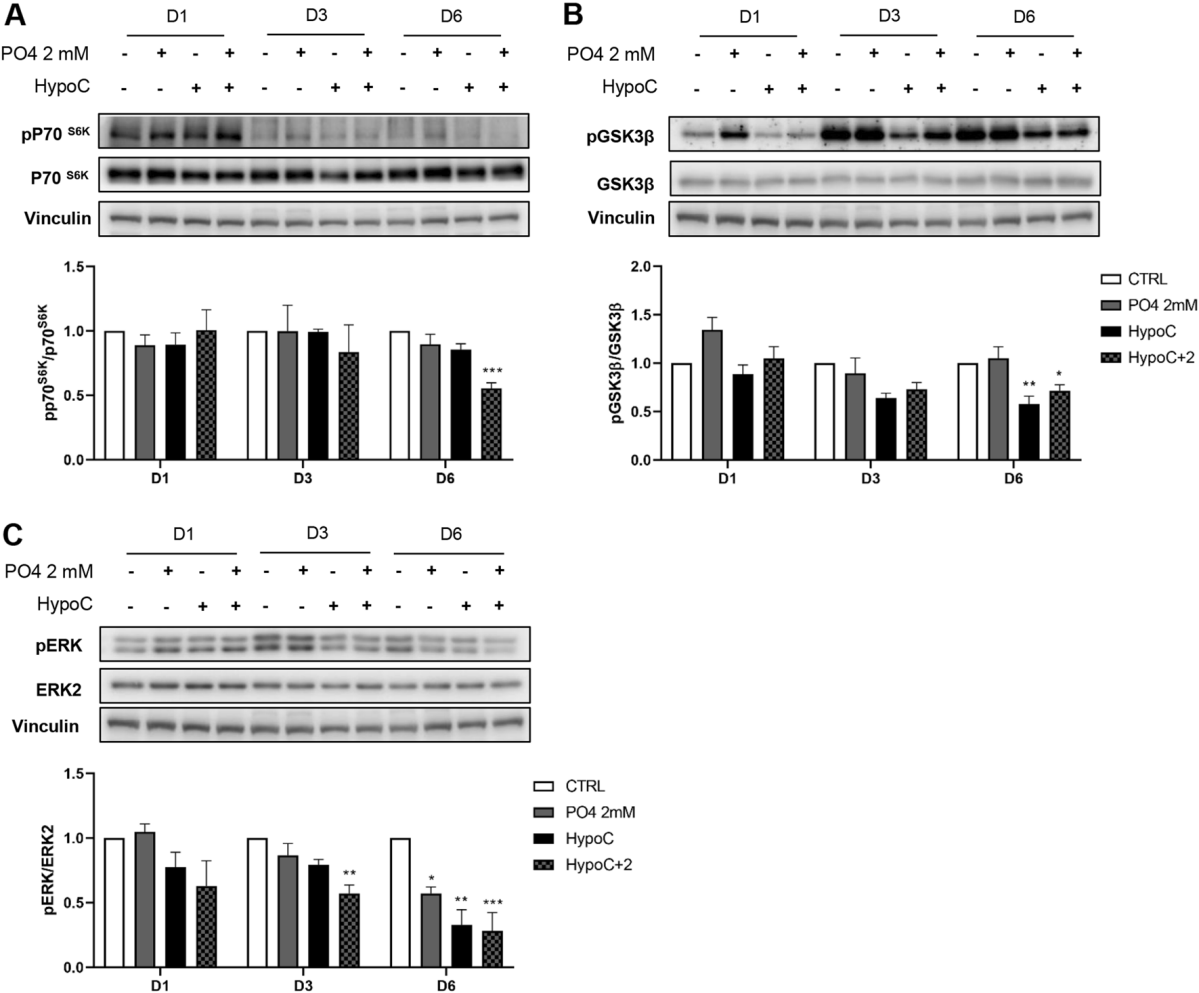
Effect of hypocalcemia and hyperphosphatemia on IGF-1 pathway in vitro. Representative blot which depicts p70^S6K^ and its phosphorylation pp70^S6K^ (**A**), GSK-3β, and its phosphorylation pGSK-3β (**B**), ERK and its phosphorylation pERK1/2 (**C**) after 1–3–6 days of differentiation. Vinculin was used as an internal control. (*p < 0.05; **p < 0.01 vs*.* CTRL)

Extracellular hypocalcemia associated with severe hyperphosphatemia significantly downregulated p70^S6K^ phosphorylation, a downstream kinase of IGF-1, after 6 days of cell differentiation (0.5 ± 0.03-fold decrease vs CTRL), as depicted in Fig. 5A.

Furthermore, we evaluated another downstream pathway of IGF-1, the glycogen synthase kinase-3β (GSK3β), which is usually phosphorylated; its activity was inhibited in muscle hypertrophy conditions and therefore β-catenin was not degraded [26, 27]. The HypoC in combination with hyperphosphatemia induced downregulation of GSK3β phosphorylation (0.6 ± 0.09 and 0.7 ± 0.07-fold decrease, respectively, vs. CTRL (Fig. 5B).

### Effects of extracellular low calcium level in combination with hyperphosphatemia on ERK phosphorylation

Previous data demonstrated that ERK2 is required for the multinucleated myofibers formation, since its absence leads to slower growth, altered contractility, and protein synthesis [28]. Thus, we also evaluated the involvement of this kinase in the events observed in extracellular ion alteration. Interestingly, the PO4 2 mM condition induced a 0.6 ± 0.06-fold decrease, the Hypo condition induced a 0.6 ± 0.05-fold decrease, and the Hypo + PO4 (either 0.5 or 2 mM) condition induced 0.3 ± 0.14-fold decrease in ERK phosphorylation compared to control cells (Fig. 5C).

## Discussion

The results of our study demonstrate that extracellular low concentrations of calcium in the presence of high concentrations of PO4 in an in vitro model, mimicking a clinical condition of hypoparathyroidism, compromises the differentiation pattern of muscle cells. Indeed, it is well known that hypoparathyroidism induces evident clinical symptoms related to muscle alteration, such as cramps or paresthesia. The presence of these symptoms has been correlated with the acute effect of hypocalcemia on muscle contractility, which is typically managed over the long term with calcium supplementation and active vitamin D analogs, facilitating ion absorption. Nevertheless, some evidences suggest that even in patient groups achieving target calcium levels through adequate supplementation, there may persist neuromuscular symptoms, which are associated with a reduced quality of life compared to healthy controls [29].

Hyperparathyroidism thus presents itself as a complex condition that cannot be solely attributed to the treatment of hypocalcemia. However, the cellular mechanisms underlying are not still fully clarified. Thus, in an attempt to characterize the potential mechanisms causing these events, we have re-created an in vitro condition/setting resembling hypoparathyroidism, by supplementing medium with low concentrations of calcium and high concentrations of PO4 with respect to standard growth conditions.

The lack of PTH is reported to induce the onset of symptoms associated with persistently low blood calcium levels (hypocalcemia) and elevated phosphates (hyperphosphatemia). Patients affected by hypoparathyroidism show alteration in bone microarchitecture and bone mass, as well as skeletal muscle complications characterized by reduction of muscle strength, muscle dysfunction, and myopathies, which could be responsible for an increased risk of instability leading to falls and fractures, as suggested by other studies [5, 30]. Although the effect of PTH on bone is well established, the effects of PTH, or the lack of it, on skeletal muscle are still not fully known [5].

Skeletal muscles are indispensable for vital functions, such as movement, postural maintenance, respiration, and thermogenesis. Skeletal muscle tissue is mainly made up of postmitotic multinucleated fibers, and by a pool of satellite cells responsible for maintaining the muscle tissue function [31]. Among the satellite muscle cells, there are the satellite stem cells, which, in response to specific stimuli (i.e., physical trauma or growth signals), give rise to cohorts of committed and therefore progenitor satellite cells. Myogenic progenitors proliferate and, if necessary, differentiate through fusion to restore fiber integrity and function [31].

Myogenic regulatory factors (MRFs) are a family of transcription factors that regulate the fate of an entire muscle cell lineage. These factors are represented by MyoD, Myf5, myogenin, and myogenic regulatory factor 4 (MRF4), responsible for the regulation of proliferation, differentiation, and the assembly of the sarcomere [32]. MyoD is necessary for cells’ commitment to the myogenesis program, while myogenin takes part in the execution of the differentiation [33]. Moreover, another crucial marker in muscle cell differentiation is MHC, a late differentiation marker and it is part of the sarcomeric structure of skeletal muscle [34]. A recent article from Raimann et al. [16] showed that the exposure of muscle cells to high levels of phosphate induced an impairment of differentiation process, by reducing MyoD, myogenin, and MHC expression.

Calcium has a pivotal role in muscle contraction and Romagnoli et al. suppose that an alteration of the serum calcium concentration can influence the skeletal muscle tissue [5, 35].

Additionally, Romagnoli et al*.* also showed that the exposure of skeletal muscle satellite cells isolated from human biopsies (hSCs) to PTH (1–84) for 30 min induced a myogenic differentiation without altering cell proliferation [36]. The authors speculated that PTH could be a new therapeutic strategy for the regulation of different skeletal muscle processes in patients affected by hypoparathyroidism and showing muscle dysfunction [36]. In accordance with the role of PTH on improvement of myogenic differentiation through maintenance of extracellular ion concentration, in our study, we simulated the effect of low serum PTH. Indeed, our data show that the exposure of C2C12 cells to a condition of extracellular hypocalcemia associated with hyperphosphatemia induced a decrease of MyoD and myogenin even after 1 day of differentiation. Moreover, there was a decrease in mRNA expression of MyHC I, MyHC IIa, and MyHC IIb as well as a decrease in protein expression of MHC in cells exposed to PO4 and low calcium compared to control cells. Our data support the hypothesis that low calcium in combination with hyperphosphatemia induces an alteration in muscle differentiation compromising the health of the muscle.

A very important hormone for muscle function and homeostasis is IGF-1. This protein is mainly produced by the liver and plays a very important role in the regulation of both anabolic and catabolic pathways in skeletal muscle [20, 21]. It IGF-1 is important in the regulation of protein synthesis in skeletal muscle via the PI3K/Akt/mTOR and PI3K/Akt/GSK3β pathways [20–23]. Akt phosphorylates and inhibits tuberous sclerosis 1 and 2 (TSC1/TSC2) and activates a protein Ras homolog enriched in the brain (Rheb), which in turn activates the mTOR complex-1 (mTORC1), resulting in phosphorylation of p70^S6K^ involved in the regulation of protein synthesis [20].

Furthermore, IGF-1 is involved in skeletal myogenesis and is associated with muscle mass, strength, and in the proliferation of muscle satellite cells (MSCs) [36]. Indeed, during the differentiation stage, the IGF-I and IGF-II mRNA levels increase [37].

Interestingly, our data showed that low calcium condition in combination with severe hyperphosphatemia induced a decrease in IGF-1 without modulating IGFR1 mRNA expression. Moreover, the same extracellular condition influenced the signaling cascade downstream IGF1. After 6 days of differentiation, downregulation of p70^S6K^ and GSK3β phosphorylation were observed, suggesting an impairment of global, skeletal muscle protein synthesis.

We also observed another crucial hormone in skeletal muscle cell homeostasis, the irisin.

This hormone derives from the proteolytic cleavage of FNDC5 extracellular fragment and secreted in the blood flow [25]. The injection of irisin into a damaged muscle of mice enhanced regeneration and induced hypertrophy, likely due to the activation of satellite cells, increase of protein synthesis, and reduction of protein degradation [38, 39]. Our results show a decreased expression level of FNDC5, the gene coding for irisin induced by the altered extracellular ions condition, strongly suggesting that alteration of this hormone level might further contribute to the muscle damage observed in patients affected by hypoparathyroidism.

Further, low calcium, in combination with severe or moderate hyperphosphatemia, induced downregulation of ERK2 phosphorylation, which is required for multinucleated myofiber formation [28]. Therefore, our data lead to hypothesize that hypocalcemia in combination with hyperphosphatemia might also induce a possible increase in muscle damage, likely altering muscle transcription factors as well as growth factors important for muscle differentiation and function.

In conclusion, the results of our study show that a condition of extracellular hypocalcemia plus hyperphosphatemia in vitro induces a decrease of muscle cell differentiation, confirmed by a reduction of multinucleated fibers. Moreover, these results may also suggest that the observed effects are induced by IGF-1 reduction, by irisin decrease and by inactivation of the main pathways involved in skeletal muscle protein synthesis.

Finally, we conclude that these findings may highlight previously less-understood pathogenic mechanisms that can further impair muscle cell function and indirectly contribute to the persistence and challenging management of symptoms in individuals with hypoparathyroidism.
